# Evaluation of Epic® label-free technology to quantify functional recombinant hemagglutinin

**DOI:** 10.1186/s12575-015-0019-5

**Published:** 2015-03-09

**Authors:** Lianlian Jiang, Maryna C Eichelberger

**Affiliations:** Division of Viral Products, Office of Vaccines Research and Review, Center for Biologics Evaluation and Research, Food and Drug Administration, 10903 New Hampshire Avenue, Silver Spring, MD 20993 USA

**Keywords:** Influenza, Hemagglutinin, Label-free technology, Potency, Fetuin, Corning, Epic, Enspire, Recombinant

## Abstract

**Background:**

Alternative methods are being sought to measure the potency of influenza vaccines. Label-free technologies that do not require the use of hemagglutinin (HA)-specific antisera are particularly attractive as the preparation of antiserum delays availability of potency reagents. The objective of these experiments was to evaluate the use of a Corning Epic® label-free method to quantify functional influenza hemagglutinin in rHA preparations. The method was optimized to quantify recombinant HA (rHA) of B/Brisbane/60/2008 (B/BR/08). Fetuin was immobilized onto plates and the change in wavelength of refracted light measured using an Enspire (Perkin Elmer) instrument.

**Results:**

The change in wavelength measured in response to addition of rHA of B/BR/08 was proportional to its concentration and was optimal in the presence of native rHA conformations. However, the assay was strain-dependent and did not correlate with HAU measured using turkey red blood cells.

**Conclusions:**

The Corning Epic® label-free method is suitable for quantifying the native forms of rHA for B/BR/08 and A/Brisbane/59/2007 (H1N1) and A/Hangxhou/3/2013 (H7N9). This method is a useful tool for research purposes but further investigation is needed to identify suitable glycoproteins to use as ligands that allow quantification of HAs from a broader range of virus strains.

**Electronic supplementary material:**

The online version of this article (doi:10.1186/s12575-015-0019-5) contains supplementary material, which is available to authorized users.

## Background

The potency of influenza vaccines is currently measured by single radial immunodiffusion (SRID) assay [1]. In this method, antigenically-intact antigen passively diffuses through agar containing monospecific sheep antibodies until a critical concentration is reached at which a precipitant forms. The precipitant is stained and the diameter of the zone measured to quantify the amount of antigen. SRID is suitable as a potency assay because it clearly distinguishes between antigen that induces hemagglutination inhibiting (HAI) antibodies and denatured antigen that induces poor HAI titers, however, the antiserum needed for this assay can take many weeks to produce, particularly when there are difficulties in purifying the HA immunogen. Since this bottleneck could impact timely availability of seasonal and pandemic influenza vaccines, alternative potency assays that do not require the use of antisera are being sought [2].

Label-free biosensing technologies with optical detection platforms, such as surface plasmon resonance (SPR), bio-layer interferometry and Corning’s Epic® technology, have been used to quantify and determine avidity of many biomolecular interactions [3-6]. In most instances, the interactions between small molecules are investigated to elucidate relative binding of ligands and receptors or to identify inhibitors of these interactions, although the Epic® technology is also used to identify mass distribution within cells [4]. The ease of measuring interactions of the native molecules without the use of labeled antigen-specific antibodies or other staining techniques, make label-free systems ideal for also investigating functional interactions between large multimeric glycoproteins and cellular receptors.

The trimeric form of HA that is required to induce functional antibodies is also the form needed to bind sialic acid-containing receptors [7,8]. Schofield and Dimmock reported the use of SPR to measure the interaction between whole influenza virus and antibody [9] and Hidari et al., quantified the functional receptor binding property of HA using ganglioside-coated chips [10]. Others have demonstrated that receptor binding can be accomplished using chips coated with large glycoproteins such as fetuin as well as chemically synthesized biotinylated multivalent glycans [11]. Since the ability of HA to bind to receptors requires it to have native trimeric conformation, and the native structure of HA corresponds to the antigenic form needed to induce HA inhibiting antibodies, SPR assays have been designed to measure influenza vaccine potency [12].

Like SPR, the Corning Epic® technology is a label-free technology but it differs from SPR in that the Epic® reaction does not take place under flow conditions and the read-out is different. Epic® technology employs resonance wave gating to measure the change in wavelength of refracted light rather than a change in reflected light energy that is absorbed by a gold sensor. The Enspire Multimode plate reader (Perkin Elmer, Waltham, MA) is a benchtop multimode instrument that includes the Corning Epic® label-free technology which allows measurement of changes within a cell as well as measurement of biochemical interactions. We previously demonstrated that influenza infection of Madin-Darby canine kidney cells resulted in a signal measured by Epic®, providing a potential high throughput tool to screen for influenza antivirals [13]. Since this technology also has capacity to record changes due to binding events, this report explores its use for quantifying the interaction between recombinant HA (rHA) and receptors present on a large glycoprotein, fetuin.

The ability of Epic® label-free technology to quantify the native form of rHA was tested by measuring its interaction with fetuin. Since HA binding depends on the presence of sialic acid, specificity was demonstrated through the use of asialofetuin as a control. Binding of a number of different rHAs from seasonal and pandemic viruses was examined, demonstrating that while the assay is suitable for quantifying some rHAs, the HAs of several influenza viruses had no (or little) reactivity with fetuin.

## Results and discussion

### Optimization of conditions for quantitation of HA-receptor binding by label-free Epic®

**Figure 1:**
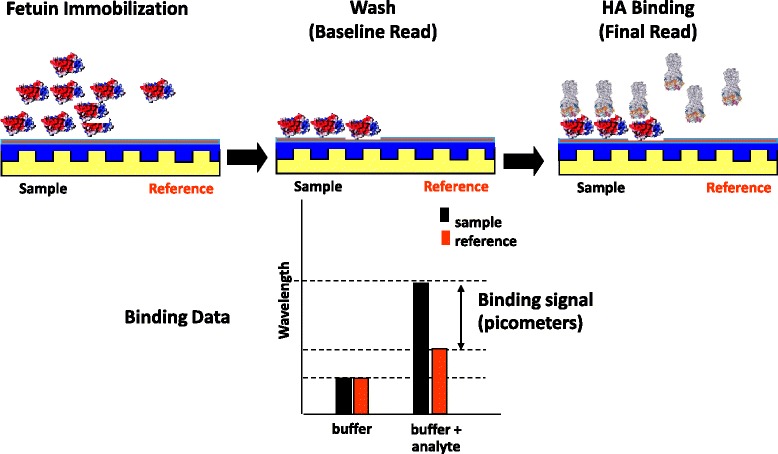
**Steps in the Epic® assay for quantification of recombinant HA.** Immobilization of fetuin; wash step and baseline read of refracted light wavelength; addition of HA and final read of refracted light wavelength. The response is measured as the difference in wavelength, measured in picometers (pm).

The steps conducted to quantify ligand (rHA) with capacity to bind receptor (sialic acid containing carbohydrates on fetuin) are shown in Figure 1: (i) fetuin immobilization step: this is the chemical conjugation of the receptor-containing glycoprotein to pre-activated plates; (ii) wash step: following wash and equilibration in the ligand buffer, the plate is read to establish a baseline reading; (iii) HA binding step: the ligand (rHA) is added and the wavelength of refracted light is measured by a sensor in the portion of the well containing immobilized fetuin and an internal control sensor located in a portion of the well that has no capacity to immobilize fetuin. The difference in wavelength (measured in picometers (pm)) is reported as the response.

**Figure 2:**
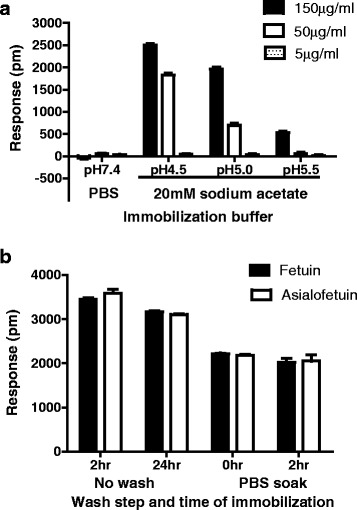
**Determination of optimal conditions for fetuin immobilization. (a)** Immobilization of different fetuin concentrations in different pH solutions shows maximum immobilization with 150 μg/ml at pH 4.5; **(b)** Baseline read of fetuin and asialofetuin after 2 hr and 24 hr immobilization are similar; baseline read immediately after washing wells (0 hr) or after washing and 2 hr equilibration (soak step) were similar. The wells that were washed contained fetuin/asialofetuin immobilized for 24 hrs.

To identify the optimal concentration and pH for fetuin immobilization in the first assay step, various fetuin dilutions (150, 50 and 5 μg/ml) were prepared in 20 mM sodium acetate pH 4.5, 5.0 and 5.5. The change in wavelength measured after immobilization was proportional to the ligand concentration and was greatest in 20 mM sodium acetate pH 4.5 (Figure 2a). Similar signals (change in wavelength of ~2000 pm) were obtained with asialofetuin. After washing the plate immobilized with 150 μg/ml fetuin or asialofetuin, the response decreased (Figure 2b) indicating that this concentration was in excess of the amount required for maximal chemical conjugation. Responses of ~2000 pm are generally used in other applications of Epic® and therefore the response obtained with fetuin/asialofetuin was considered adequate for further experiments.

**Figure 3:**
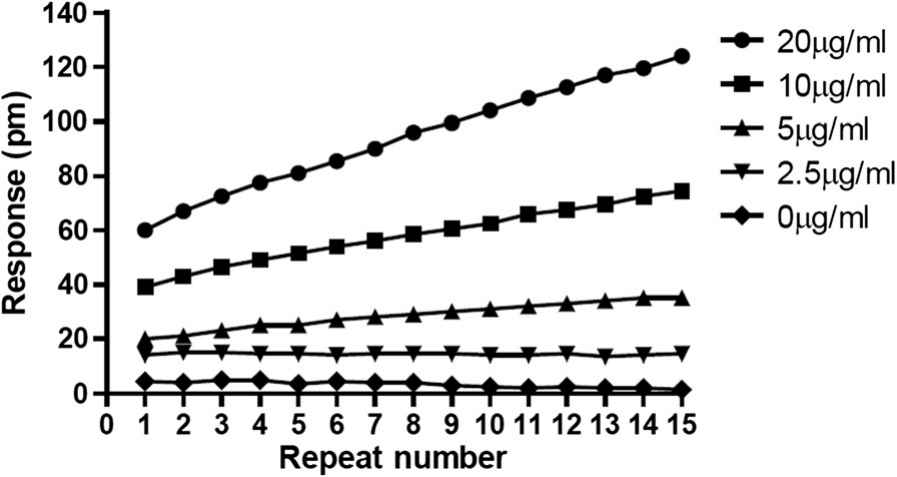
**Response measured after addition of increasing concentrations of rHA increases with time.** rHA of B/BR/08 was added to wells containing immobilized fetuin and change in wavelength read at 2 min intervals over a 30 min time period. The greatest difference in response to different rHA concentrations was observed at 30 min.

The remaining assay steps were conducted following the manufacturer’s guidance as described in the Methods section, with a baseline wavelength reading recorded after 4 hr equilibration of the fetuin-immobilized plate in the ligand buffer (PBS, pH 7.4). Serial dilutions of rHA of B/Brisbane/60/2008 (B/BR/08, Protein Sciences, Meriden, CT) were added to quadruplicate wells containing fetuin immobilized at various concentrations. The wavelength of refracted light was read soon after the addition of rHA and over 30 min at 2 minute intervals because it was expected that equilibrium (and therefore maximum signal) may not be reached immediately. The instrument reported the difference in wavelength from the sensor in the portion of the well containing immobilized fetuin and the internal control sensor without immobilized fetuin. As shown for wells containing immobilized fetuin at 150 μg/ml, the change in wavelength (response) increased over time when 5, 10 and 20 μg/ml rHA was added, however the differences were fairly proportional over time suggesting that the response measured immediately after addition of rHA would give similar results to responses measured after the longest time interval tested (Figure 3). The difference in response observed by addition of increasing amounts of rHA even at the initial reading was indicative that there was significant rHA binding to fetuin in a dose-dependent manner by the time the plate was placed in the reader. The response did not increase over time with 2.5 μg/ml rHA indicating that at this low concentration high avidity interactions between rHA and fetuin were complete by the time the plate was put in the reader. Additional experiments were conducted in which the response was measured after a longer period (Additional file 1: Figure S1a-d); the signal due to rHA binding increased slightly with time, but the difference was small and did not warrant an incubation period after addition of rHA prior to measuring the response.

### The response measured by Epic® label-free assay is specific for rHA binding to fetuin

**Figure 4:**
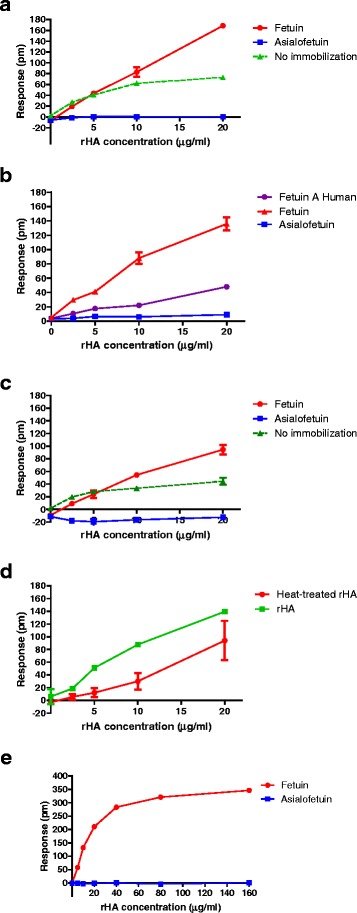
**Specificity of the Epic® response. (a)** Addition of increasing rHA concentrations to empty wells (green line) or wells containing immobilized bovine fetuin (red line), or asialofetuin (blue line); **(b)** Addition of rHA to wells containing immobilized human fetuin (purple line), asialofetuin (blue line) or bovine fetuin (red line). Human fetuin and asialofetuin were immobilized at 20 μg/ml; bovine fetuin was immobilized at 150 μg/ml; **(c)** Addition of native rHA (green line) or rHA that had been heated at 95°C for 5 min (red line) to fetuin immobilized wells; **(d)** Response of increasing rHA concentrations added to empty wells (green line) or wells containing immobilized bovine fetuin (red line) or asialofetuin (blue line) after a wash and equilibration step; **(e)** Determination of the linear working rHA concentration range. rHA of B/BR/08 was added to wells containing immobilized fetuin (red line) or asialofetuin (green line) at 5, 10, 40, 80 and 160 μg/ml. In all experiments except those using human fetuin, bovine fetuin and asiaolofetuin were immobilized at 150 μg/ml under optimal conditions. In experiments using human fetuin, immobilization was performed with glycoproteins at 20 μg/ml.

Initial experiments were confounded by results showing responses after adding rHA to wells that did not contain fetuin (wells without immobilized protein). Obtaining a signal that was clearly non-specific in empty wells emphasized the importance of comparing changes in wavelength for wells containing immobilized receptor and a suitable control glycoprotein. Since HA binds to α2-3 and α2-6 sialylated glycoproteins we tested asialofetuin as a negative control. When asialofetuin and fetuin were added to chemically-activated plates at the same concentration, the signal obtained was similar, indicating that they achieved a comparable degree of immobilization (Figure 2b). Incubation of rHA in wells containing immobilized asialofetuin resulted in minimal changes in wavelength (response) compared to the response observed in wells containing immobilized fetuin (Figure 4a-e and Additional file 1: Figure S1 and Additional file 2: Figure S2). This result demonstrates that the response measured when rHA was added to fetuin-immobilized wells was indeed due to specific binding of rHA to sialic acid-containing receptors. Asialofetuin was therefore used as negative control in all experiments.

To verify that the response measured was not the result of rHA chemically reacting with the plate (becoming immobilized to wells), the chemical reaction was prevented by treatment of the wells with 200 mM ethanolamine before addition of rHA. This treatment reduced the binding of HA to fetuin (Additional file 2: Figure S2), suggesting that it had a detrimental impact on the receptor structure. Blocking the chemically-reactive sites in empty wells did not eliminate the response (Figure 4a), supporting the hypothesis that rHA was not reacting with the plate. The change in wavelength following addition of rHA to empty wells was most likely an indication of differences in refractive index of the solution with increasing rHA concentration. Since blocking the chemically-reactive groups in each well did not benefit the measurement of the specific response and specificity of the response is demonstrated in each assay by inclusion of asialofetuin as control, the blocking step was omitted from the general protocol.

To evaluate whether the signal could be improved by removing unbound rHA and to confirm that the signal was indeed due to binding of rHA to receptors on fetuin, the plate was washed after addition of rHA. Washing the plate did reduce the response to some degree (Figure 4d), suggesting that some rHA interactions with fetuin had low avidity, however the signal was primarily retained. Since the response to asialofetuin served as a negative control, a wash step was not warranted for routine experiments.

To investigate whether the signal could be increased by using fetuin of human origin, the response was measured in wells containing immobilized human recombinant fetuin A (Sigma, St Louis, MO). This recombinant was purified from a mammalian (HEK293 cells) expression system. Due to the expense of this product, a low concentration was immobilized onto the plate. There was good sensitivity in measuring a response of rHA binding to human fetuin (response observed even with 2.5 μg/ml rHA), however the signal was significantly less than the response measured for rHA binding to immobilized bovine fetuin (Figure 4b). Further experiments therefore continued to measure rHA binding to bovine fetuin.

### Quantification of rHA in the Epic® label-free assay is strain dependent

**Figure 5:**
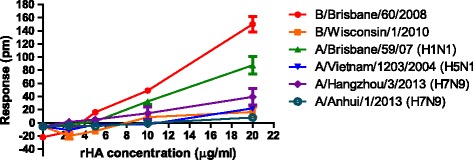
**Epic® response is strain-dependent.** The response was measured after addition of increasing concentrations of rHA from different influenza A and B strains. The ability of each rHA to agglutinate turkey red blood cells is reflected in the HAU provided after each strain name: B/Wisconsin/1/2010 (1024 HAU/μg), B/BR/08 (2048 HAU/μg), A/Brisbane/59/2007 (H1N1) (2048 HAU/μg), A/Vietnam/1203/2004 (H5N1) (1024 HAU/μg), A/Anhui/1/2013 (H7N9) (4 HAU/μg) and A/Hangzhou/2013 (H7N9) (32HAU/μg). The assays used bovine fetuin immobilized under optimal conditions at 150 μg/ml. The results of binding to asialofetuin were all negative (no response after addition of rHA).

Influenza vaccine potency assays should ideally be generally applicable so that the same method can be used to quantify antigenic forms of rHA from both influenza A and B viruses. To determine whether the conditions established for quantifying the rHA of B/BR/08 were applicable also to other influenza B viruses and influenza A H1N1, H5N1 and H7N9 viruses, the change in wavelength was measured after adding rHA from a variety of strains to fetuin and asialofetuin-immobilized wells. To demonstrate that the rHAs retained native conformation, we determined hemagglutination units (HAU) using turkey red blood cells. The rHA of B/BR/08, B/Wisconsin/1/2010, A/Brisbane/59/07 (H1N1) and A/Vietnam/1203/2004 (H5N1) had ~1024 HAU/μg; while the rHA of A/Hangzhou/3/2013 (H7N9) and A/Anhui/1/2013 (H7N9) had only 32 and 4 HAU/μg, respectively. The low level of agglutination by H7 proteins may indicate low binding avidity to receptors on turkey red blood cells rather than evidence of HA denaturation. Addition of each of the rHA to bovine fetuin-immobilized wells did not yield a uniform response; the greatest response was observed with rHA from B/BR/08, with ~140 pm at 20 μg/ml; the response measured after addition of rHA from A/Brisbane/59/2007 (H1N1) was less, with ~80 pm at 20 μg/ml (Figure 5). Responses did not reflect HAU and were not uniform for each type/subtype of virus; for example, the response to rHA of B/Wisconsin/1/2010 was minimal while the rHA of B/BR/08 resulted in reproducible dose-dependent responses. Also, even though the HAU of the rHA preparation of A/Hangzhou/3/2013 (H7N9) was low, there was a measurable response (~80 pm at 20 μg/ml).

### The Epic® label-free assay quantifies functional rHA of B/BR/08 over a range suitable for potency testing

Since potency test results should be a measure of protein antigenic form, experiments were designed to evaluate the ability of the Epic® assay to discriminate between native and denatured rHA. Red blood cell (RBC) agglutination provides evidence that rHA is in its native trimeric form [14], and therefore the response for rHA of B/BR/08 that has measurable HAU was compared to the response measured for the same rHA preparation that had been heated to 95°C for 5 min and no longer agglutinated RBC. The HAU for each preparation was 2048 and 0 respectively. Titration of each rHA preparation showed that the response was greatest when the native structure was present, although rHA did retain some ability to interact with fetuin after heat denaturation (Figure 4c). Given that several rHAs did not bind to fetuin (Figure 5), the Epic® response is unlikely to be due to a non-specific interaction between HA and fetuin. Instead, the response observed with heat-treated B/BR/08 may indicate that trimeric forms of rHA that can bind fetuin but are not in sufficient quantity to agglutinate RBC, remain in the preparation. Unfortunately, the amount of rHA available was too little to evaluate the extent of denaturation by independent methods. Fluorescence spectral analysis of HA of a different influenza B virus showed that incubation at 60°C for 7 days resulted in partial denaturation [15], suggesting the feasibility of retaining some HA with ability to bind receptors after 5 minutes of heating at 95°C. Additional immunogenicity or SRID assays are needed to establish whether this is indeed the case. Nevertheless, the results show that an optimal Epic® response is obtained when functional HA is used in the assay, and suggests that the Epic® assay may be suitable for determining the potency of this rHA.

It is important that a reasonable range of HA concentrations can be quantified in any new potency assay. Since Epic® does not discriminate between influenza A subtypes, nor influenza types A and B, this assay would only be useful for determining the potency of monovalent vaccines or monovalent bulk lots. Titration of B/BR/08 rHA on immobilized fetuin suggests the receptors are saturated at rHA concentrations >40 μg/ml (Figure 4e), but that there is good linearity of the response with increasing concentrations between 2.5-20 μg/ml. These results suggest that the concentration of native rHA in unknown samples or the stability of a rHA preparation could be measured in an Epic® assay in which a standard curve of the response is generated for a reference rHA preparation between 2.5 – 20 μg/ml; the concentration of an unknown sample would then be calculated from this standard curve after measuring the response at several sample dilutions.

### Application of Epic® label-free quantification to research

Our results suggest that the Epic® label-free method using fetuin-immobilized plates is suitable for quantifying the native trimeric form of rHA of B/BR/08, A/Brisbane/59/07 (H1N1) and A/Hangzhou/3/2013 (H7N9). It may also be suitable for the rHAs of strains that were not included in this study. Our experience shows the importance of including immobilized asialofetuin as a negative control to demonstrate specificity of the reaction in each assay. In addition, controls such as heat-denatured rHA should be included to claim conformation-dependence of the response.

The Epic® assay described in this report is somewhat more sensitive than the traditional SRID potency assay; the limit of detection (LOD) of Epic® is approximately 2.5 μg/ml (Figure 3, and Additional file 1: Figure S1 and Additional file 2: Figure S2) and the LOD of SRID is approximately 8–10 μg/ml [16,17]. Although this would allow potency testing at the level needed for current vaccine formulations, adjuvanted influenza vaccines that are dose-sparing are being developed and therefore there is a need for more sensitive potency assays. ELISAs that use HA-specific monoclonal antibodies to capture antigen have excellent sensitivity and range (this depends on monoclonal antibody and antigen, but can be used to quantify as little as 26 ng HA/ml [16]). ELISAs in which glycans are used to capture HA before addition of monoclonal antibodies to detect the bound HA are also very sensitive (reported as low as 100 ng HA/ml [18]). SPR methods that report a signal based on binding to sialylated glycoproteins have similar a LOD that is similar to that of the Epic® assay [19], however when synthetic α2-3, or α2-6 sialic acid glyans are used in SPR, the higher affinity interactions appear to increase assay sensitivity; as a little as 0.33 μg HA/ml can be quantified in these assays that have excellent dynamic range [12].

While the use of subtype-specific antisera allow SRID and ELISAs to quantify HA of a specific virus strain even when it is mixed with other HA subtypes in a trivalent or quadrivalent influenza vaccine formulation [16,20], Epic® and SPR assays that are antibody-independent do not discriminate between HA strains [12]. The latter assays are therefore only useful for determining the potency of monovalent bulk material or HA content of monovalent vaccines. While the ability to measure potency of multivalent formulations is an advantage of antibody-dependent assays, the low throughput of SRID assays make it cumbersome to perform large number of assays on a single day, whereas ELISA and Epic® assays that are conducted in a plate format are easy to perform using multichannel pipettes and automated plate readers to easily quantify HA in large numbers of samples.

The inability of the current Epic® assay to consistently measure responses for different rHAs suggests that the current assay is best suited to quantify rHAs for research purposes and not for general potency testing of vaccines. The current results align with those obtained using fetuin as a ligand in SPR that indicate this glycoprotein does not provide receptors to allow quantification of HA from all influenza viruses. While short synthetic glycoforms with α2-3 and α2-6 sialic acids are suitable ligands to measure potency of HA in vaccines by SPR [12], short glycans did not immobilize efficiently to the chemically-activated plates used for Epic® analysis (results not shown). Further testing is needed to identify glycoproteins that can be used to quantify HAs from a broad range of influenza viruses in the Epic® label-free method.

## Conclusions

In this report we describe the optimization of an Epic® label-free assay to quantify the native form of rHA of B/BR/08. Immobilized bovine fetuin provides a source of receptors to which functional HA binds; wells containing immobilized asialofetuin are used as a negative control. Further experiments are needed to determine the comparability of results measured by Epic® for this antigen and the standard influenza SRID potency assay. The specificity of rHA from different influenza viruses is not uniform for fetuin and therefore broader application of the Epic® assay will require additional testing to identify immobilized substrates to which HA from a broad range of influenza viruses can bind.

## Methods

### Reagents

rHAs of influenza A (H1N1 and H5N1) and B viruses were purchased from Protein Science Inc (Meriden, CT) and rHAs of influenza A H7N9 viruses were purchased from Sino Biological (Beijing, China). Bovine and human fetuin, asialofetuin and all other chemicals were purchased from Sigma (St Louis, MO).

### Measurement of hemagglutination units (HAU)

rHA was serially diluted in PBS in a 96 well round bottom plate. An equal volume (50 μl) of 0.5% turkey red blood cells was added to each well, mixed gently and allowed to settle for 45 min at room temperature. The lowest concentration of rHA that resulted in agglutination was recorded as one HAU.

### EPIC® assay for determination of functional rHA concentration

The assay steps are shown in Figure 1. Epic® plates (384 well) that were chemically activated to allow protein immobilization were purchased from Perkin Elmer (Waltham, MA). Fetuin and asialofetuin were immobilized by adding 15 μl of ligand (150 μg/ml) in 20 mM sodium acetate pH 4.5 using a multi-channel pipette and incubating at room temperature for 1 hr. Initial experiments used plates that had been stored overnight at 4°C however this additional incubation was not necessary to achieve optimal immobilization. In some experiments the microplates were blocked from further chemical reactions by incubation with 200 mM ethanolamine in 150 mM borate buffer (pH9.2) for 15 minutes. Plates were then washed three times by applying 25 μl assay buffer (PBS, pH 7.4). A final volume of 15 μL assay buffer was added and any potential air bubbles removed by centrifugation of the plate (400 rpm x 1 min). The plates were equilibrated by incubating at room temperature for 4 hr. The change in signal due to immobilization was recorded by comparing wavelength of light refracted from the immobilized surface and a reference surface within the same well on an Enspire® multimode plate reader (Perkin Elmer). Serial dilutions of rHA (15 μl) in dilution buffer were then added to quadruplicate wells and the change in wavelength recorded 15 times at 2 min intervals.

## Additional files

Additional file 1:
**The label-free binding response does not require extensive incubation after addition of rHA to fetuin-immobilized wells.** After a wash and soak step, rHA was added to wells containing immobilized fetuin or asialofetuin and the change in wavelength from baseline read immediately (0 hr) and 1, 2 and 3 hrs later. Results are shown as the average of duplicate wells; standard deviation is shown by a cross-hatch bar. Additional file 2:
**The binding response is impeded if chemically-reactive sites are blocked after fetuin-immobilization.** Response of rHA binding to immobilized fetuin (red line) or asialofetuin (blue line) was measured following (A) the usual protocol (no chemical blocking) or (B) treatment of the plate with 200 mM ethanolamine in borate buffer, pH 9.5, after the immobilization step (blocking). Results are shown as the average of duplicate wells; standard deviation is shown by a cross-hatch bar.

## Abbreviations

HA: Hemagglutinin
HAI: Hemagglutination inhibition/inhibiting
pm: Picometers
rHA: Recombinant hemagglutinin
SRID: Single radial immunodiffusion
SPR: Surface plasmon resonance

## References

[1] Williams MS, Mayner RE, Daniel NJ, Phelan MA, Rastogi SC, Bozeman FM (1980). New developments in the measurement of the hemagglutinin content of influenza virus vaccines by single-radial-immunodiffusion. J Biol Stand.

[2] Hardy S, Eichelberger M, Griffiths E, Weir JP, Wood D, Alfonso C (2011). Confronting the next pandemic–workshop on lessons learned from potency testing of pandemic (H1N1) 2009 influenza vaccines and considerations for future potency tests, Ottawa, Canada, July 27–29, 2010. Influenza Other Respir Viruses.

[3] Cooper MA (2003). Label-free screening of bio-molecular interactions. Anal Bioanal Chem.

[4] Fang Y (2006). Label-free cell-based assays with optical biosensors in drug discovery. Assay Drug Dev Technol.

[5] Naik S, Brock S, Akkaladevi N, Tally J, McGinn-Straub W, Zhang N (2013). Monitoring the kinetics of the pH-driven transition of the anthrax toxin prepore to the pore by biolayer interferometry and surface plasmon resonance. Biochemistry.

[6] Sun H, Wei Y, Deng H, Xiong Q, Li M, Lahiri J (2014). Label-free cell phenotypic profiling decodes the composition and signaling of an endogenous ATP-sensitive potassium channel. Sci Rep.

[7] Khurana S, Verma S, Verma N, Crevar CJ, Carter DM, Manischewitz J (2010). Properly folded bacterially expressed H1N1 hemagglutinin globular head and ectodomain vaccines protect ferrets against H1N1 pandemic influenza virus. PLoS One.

[8] Magadan JG, Khurana S, Das SR, Frank GM, Stevens J, Golding H (2013). Influenza A virus hemagglutinin trimerization completes monomer folding and antigenicity. J Virol.

[9] Schofield DJ, Stephenson JR, Dimmock NJ (1997). Variations in the neutralizing and haemagglutination-inhibiting activities of five influenza A virus-specific IgGs and their antibody fragments. J Gen Virol.

[10] Hidari KI, Shimada S, Suzuki Y, Suzuki T (2007). Binding kinetics of influenza viruses to sialic acid-containing carbohydrates. Glycoconj J.

[11] Suenaga E, Mizuno H, Penmetcha KK (2012). Monitoring influenza hemagglutinin and glycan interactions using surface plasmon resonance. Biosens Bioelectron.

[12] Khurana S, King LR, Manischewitz J, Coyle EM, Golding H (2014). Novel antibody-independent receptor-binding SPR-based assay for rapid measurement of influenza vaccine potency. Vaccine.

[13] Wu M, Long S, Frutos AG, Eichelberger M, Li M, Fang Y (2009). Interrogation of phosphor-specific interaction on a high-throughput label-free optical biosensor system-Epic system. J Recept Signal Transduct Res.

[14] Khurana S, Verma S, Verma N, Crevar CJ, Carter DM, Manischewitz J (2011). Bacterial HA1 vaccine against pandemic H5N1 influenza virus: evidence of oligomerization, hemagglutination, and cross-protective immunity in ferrets. J Virol.

[15] Luykx DM, Casteleijn MG, Jiskoot W, Westdijk J, Jongen PM (2004). Physicochemical studies on the stability of influenza haemagglutinin in vaccine bulk material. Eur J Pharm Sci.

[16] Bodle J, Verity EE, Ong C, Vandenberg K, Shaw R, Barr IG (2013). Development of an enzyme-linked immunoassay for the quantitation of influenza haemagglutinin: an alternative method to single radial immunodiffusion. Influenza Other Respir Viruses.

[17] Eichelberger SL, Sultana I, Gao J, Getie-Kebtie M, Alterman M, Eichelberger MC (2013). Potency under pressure: the impact of hydrostatic pressure on antigenic properties of influenza virus hemagglutinin. Influenza Other Respir Viruses.

[18] Hashem AM, Gravel C, Farnsworth A, Zou W, Lemieux M, Xu K (2013). A novel synthetic receptor-based immunoassay for influenza vaccine quantification. PLoS One.

[19] Mandenius CF, Wang R, Alden A, Bergstrom G, Thebault S, Lutsch C (2008). Monitoring of influenza virus hemagglutinin in process samples using weak affinity ligands and surface plasmon resonance. Anal Chim Acta.

[20] Schmeisser F, Vasudevan A, Soto J, Kumar A, Williams O, Weir JP (2014). A monoclonal antibody-based immunoassay for measuring the potency of 2009 pandemic influenza H1N1 vaccines. Influenza Other Respir Viruses.

