# Supplementary material for: Evaluation of Epic® label-free technology to quantify functional recombinant hemagglutinin
Source: Biol Proced Online. 2015 Mar 9;17:7. doi: 10.1186/s12575-015-0019-5 (PMC4359790; doi:10.1186/s12575-015-0019-5)
Supplement: Additional file 1: — The label-free binding response does not require extensive incubation after addition of rHA to fetuin-immobilized wells. After a wash and soak step, rHA was added to wells containing immobilized fetuin or asialofetuin and the change in wavelength from baseline read immediately (0 hr) and 1, 2 and 3 hrs later. Results are shown as the average of duplicate wells; standard deviation is shown by a cross-hatch bar. [file 12575_2015_19_MOESM1_ESM.pdf]

Additional file 1

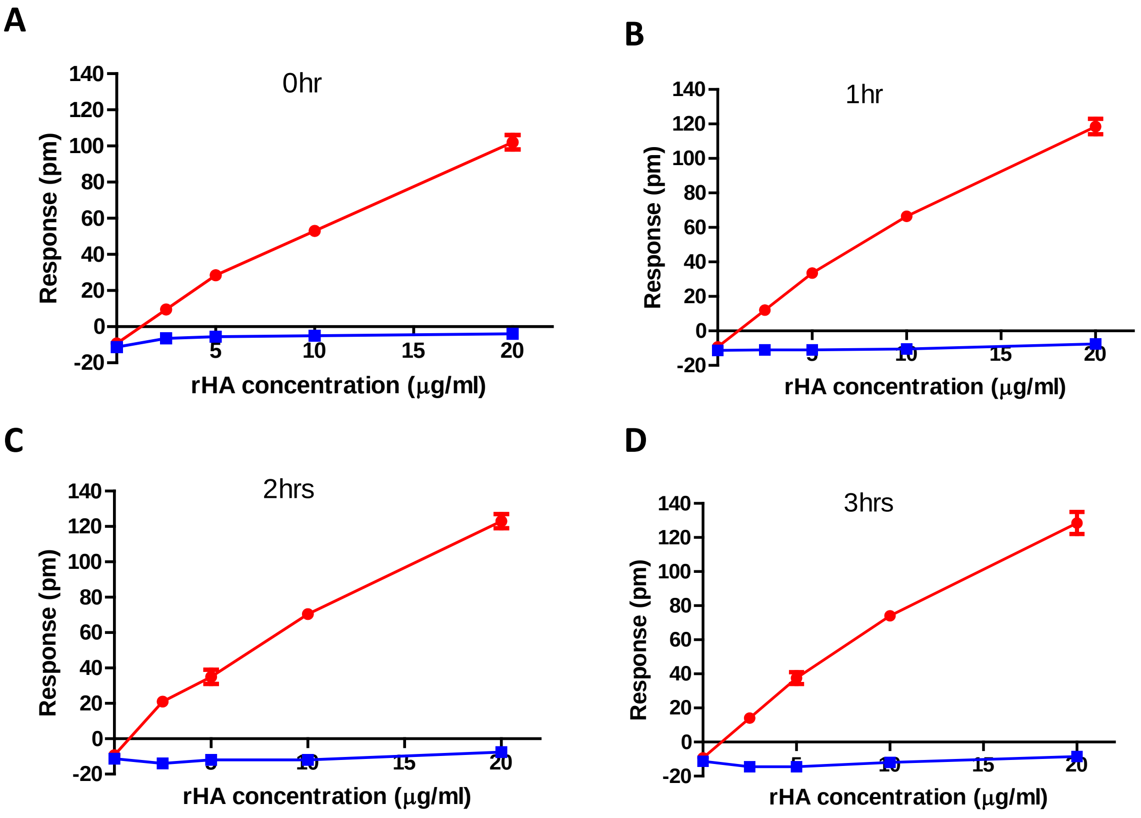

Additional file 1. The label-free binding response does not require extensive incubation after addition of rHA to fetuin-immobilized wells. After a wash and soak step, rHA was added to wells containing immobilized fetuin or asialofetuin and the change in wavelength from baseline read immediately (0 hr) and 1, 2 and 3 hrs later. Results are shown as the average of duplicate wells; standard deviation is shown by a cross-hatch bar.
