# Supplementary material for: Evaluation of Epic® label-free technology to quantify functional recombinant hemagglutinin
Source: Biol Proced Online. 2015 Mar 9;17:7. doi: 10.1186/s12575-015-0019-5 (PMC4359790; doi:10.1186/s12575-015-0019-5)
Supplement: Additional file 2: — The binding response is impeded if chemically-reactive sites are blocked after fetuin-immobilization. Response of rHA binding to immobilized fetuin (red line) or asialofetuin (blue line) was measured following (A) the usual protocol (no chemical blocking) or (B) treatment of the plate with 200 mM ethanolamine in borate buffer, pH 9.5, after the immobilization step (blocking). Results are shown as the average of duplicate wells; standard deviation is shown by a cross-hatch bar. [file 12575_2015_19_MOESM2_ESM.pdf]

Additional file 2

A

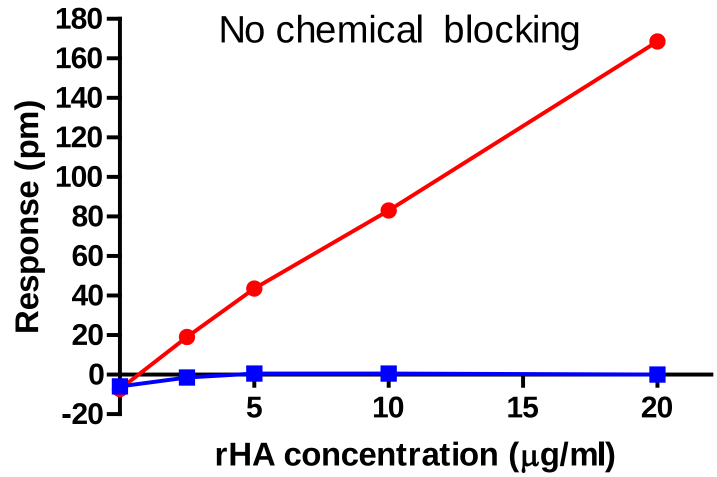

B

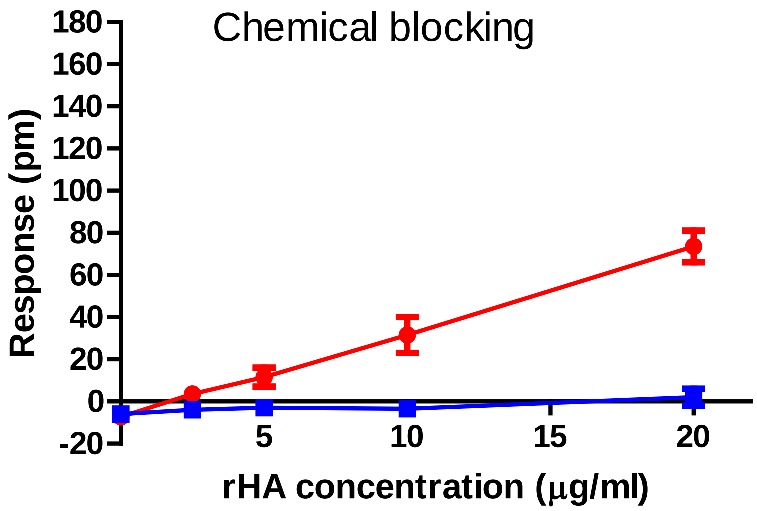

Additional file 2. The binding response is impeded if chemically-reactive sites are blocked after fetuin-immobilization. Response of rHA binding to immobilized fetuin (red line) or asialofetuin (blue line) was measured following (A) the usual protocol (no chemical blocking) or (B) treatment of the plate with 200 mM ethanolamine in borate buffer, pH 9.5, after the immobilization step (blocking). Results are shown as the average of duplicate wells; standard deviation is shown by a cross-hatch bar.
